# Invisible Color Variations of Facial Erythema: A Novel Early Marker for Diabetic Complications?

**DOI:** 10.1155/2019/4583895

**Published:** 2019-09-02

**Authors:** Victoria Blanes-Vidal, Tomas Majtner, Luis David Avendaño-Valencia, Knud B. Yderstraede, Esmaeil S. Nadimi

**Affiliations:** ^1^Group of Machine Learning, Data Science and Artificial Intelligence, Embodied Systems for Robotics and Learning (ESRL), The Mærsk Mc-Kinney Møller Institute, University of Southern Denmark, Denmark; ^2^Center for Innovative Medical Technology, Odense University Hospital, Odense, Denmark; ^3^Steno Diabetes Center, Odense University Hospital, Denmark; ^4^University of Southern Denmark, Denmark

## Abstract

**Aim:**

(1) To quantify the invisible variations of facial erythema that occur as the blood flows in and out of the face of diabetic patients, during the blood pulse wave using an innovative image processing method, on videos recorded with a conventional digital camera and (2) to determine whether this “unveiled” facial red coloration and its periodic variations present specific characteristics in diabetic patients different from those in control subjects.

**Methods:**

We video recorded the faces of 20 diabetic patients with peripheral neuropathy, retinopathy, and/or nephropathy and 10 nondiabetic control subjects, using a Canon EOS camera, for 240 s. Only one participant presented visible facial erythema. We applied novel image processing methods to make the facial redness and its variations visible and automatically detected and extracted the redness intensity of eight facial patches, from each frame. We compared average and standard deviations of redness in the two groups using *t*-tests.

**Results:**

Facial redness varies, imperceptibly and periodically, between redder and paler, following the heart pulsation. This variation is consistently and significantly larger in diabetic patients compared to controls (*p* value < 0.001).

**Conclusions:**

Our study and its results (i.e., larger variations of facial redness with the heartbeats in diabetic patients) are unprecedented. One limitation is the sample size. Confirmation in a larger study would ground the development of a noninvasive cost-effective automatic tool for early detection of diabetic complications, based on measuring invisible redness variations, by image processing of facial videos captured at home with the patient's smartphone.

## 1. Introduction

The risk of developing complications to diabetes mellitus (DM) has generally declined during the last two decades [1]. Microvascular complications include nephropathy, retinopathy, and neuropathy. Nephropathy and retinopathy will affect roughly one-third of all diabetic individuals [1, 2]. In a population-based study derived from the A1chieve study, a high level of variation in complications was found among different geographical areas [3]. Data on the prevalence of neuropathy are, however, scarce. Long-term complications of diabetes may arise in both type 1 and type 2 diabetes and in both insulin-treated and non-insulin-treated DM individuals. Diabetes complications develop gradually, and eventually, they may be disabling or even life-threatening.

Diabetic complications can affect every organ system, including the skin [4]. Cutaneous findings may be the first sign of metabolic disturbance related to undiagnosed diabetes, suboptimal management, or a prediabetic state [5]. Besides, cutaneous findings may serve as external markers of internal complications in already diagnosed diabetic patients [6, 7]. *Rubeosis faciei* has been described as a common cutaneous manifestation of diabetes mellitus [8]. The condition is characterized by a chronic facial erythema (red face). Capillary microscopy has demonstrated venular dilatation in the cheeks of diabetic patients with this condition. This venous dilatation may be related to sluggish microcirculation due to hyperglycemia [9]. The prevalence of *rubeosis faciei* reported in previous studies varies largely, from 3-7% [10, 11] to 59% [12].

*Rubeosis faciei* has been associated with three noncutaneous diabetic complications (nephropathy, retinopathy, and neuropathy) [11], possibly due to sharing microangiopathy as a common etiological factor. *Rubeosis faciei* is diagnosed during clinical examination, when the intensity of red coloration in the diabetic patient is self-evident. If *rubeosis faciei* is recognized, it should alert physicians to look for other microangiopathic complications. However, *rubeosis faciei* may go unnoticed in routine clinical practice, since the intensity of red coloration depends on the degree of vascular engorgement of the superficial venous plexus, as well as on the skin tone [9, 12].

Recently, an innovative video processing method has been presented by researchers from the Massachusetts Institute of Technology, which allows revealing tiny color changes (too small to be seen by the naked eye) in videos recorded with a regular digital camera [13, 14]. Among other medical and nonmedical applications, this method allows detecting minuscule color variations caused by blood flowing to and from the face in rhythm with the beats of the heart.

In this paper, we adapted and applied this novel color magnification method to make facial erythema (perception of venous stasis in the microcirculation) visible in videos of both diabetic and nondiabetic subjects. We then quantified this erythema, and we determined whether the intensity of this “unveiled” facial red coloration and its small variations during the blood pulse wave present specific characteristics in diabetic patients, different from those in nondiabetic subjects. Our goal is to explore whether these cutaneous variables that are not apparent to the medical professional naked eye (i.e., the invisible facial redness and the variation of this redness due to heart pulsation) could be markers of DM. The benefit of identifying novel early markers for diabetic complications using patients' videos recorded with a conventional digital camera lies in the fact that it would set the grounds for the development of a noninvasive cost-effective solution for early identification of patients who are prone to develop microvascular complications. This would, in the end, result in new healthcare strategies to reduce the burden of diabetic complications.

## 2. Research Design and Methods

### 2.1. Study Participants

Thirty subjects were enrolled in the study. The subjects formed two groups: group DM: 20 insulin-treated diabetic patients (mean ± standard deviation (SD) of age = 63 ± 10 y; 14 males/6 females) and group C: 10 control subjects (mean ± SD of age = 60 ± 6 y; 7 males/3 females). The two groups did not differ in average age and gender proportion (*p* > 0.10). Eleven DM patients were diagnosed with type 1 diabetes (mean ± SD of time from diagnosis = 28 ± 13 y), while 9 patients were diagnosed with type 2 DM (mean ± SD of time from diagnosis = 22 ± 5 y). Out of the 20 DM patients, 19 had been diagnosed with peripheral neuropathy, 19 with retinopathy, and 7 with diabetic nephropathy, and 5 of them had been diagnosed with peripheral arterial disease. Facial erythema was not apparent in any of the participants, except for one DM patient with slight facial red coloration. None of the DM patients had exhibited cranial neuropathies such as facial nerve palsy, optic neuropathy, or auditory neuropathy. Control subjects had not been diagnosed with DM, peripheral arterial disease, or any disease affecting the nervous system. The study protocol was reviewed and approved by the scientific ethical committee for Region Southern Denmark (process number: 18/297; Project ID: S-20180006). All participants were informed about the study, and signed written informed consents were obtained.

### 2.2. Experimental Setup and Video Acquisition

The experimental setup for video acquisition consisted of a laptop and a Canon EOS 1300D camera on a tripod. The camera was placed on top of the laptop screen, about 84 cm in front of the subject being examined, so that the entire face is captured. The subject was seated in a room on a comfortable chair. The experiment consisted of video recording the face of the subject for 7 min 45 s length (including two 30 s breaks), while he/she looks at the laptop monitor in front of him/her, keeping his/her head as immobile as possible. The participants were recorded twice (R1 = repetition 1, R2 = repetition 2), with a resting time between repetitions of about 10 min. The camera's focal length was 35 mm, the number of frames per second (fps) was 50, and the size of each frame was 1280 × 720 pixels. A color correction procedure was performed on the captured facial image to decrease the effect of the different environment and illumination variations, and the face tracking algorithm was used to compensate for small head movements between frames.

### 2.3. Video Processing

We first identified and trimmed the unwanted parts of the video footages, i.e., those parts that could result in inaccurate measurements of color intensity (such as the breaks in which the subject can abruptly move his head or turn it away from the camera). This resulted in three video segments (A, B, and C), with 5901, 4851, and 6251 frames each, respectively. We then applied the Eulerian video magnification (EVM) method [14, 15] to the cleansed videos. The EVM method first decomposed each frame of the input video sequence into different spatial frequency bands (spatial decomposition). We considered the time series corresponding to the value of a pixel in a frequency band and applied a bandpass filter to extract the frequency bands of interest (in the case of the variation in the redness of the face, a wide band of temporal frequencies that includes plausible human heart rates, i.e., 0.4-4 Hz). The extracted bandpass signal was multiplied by a magnification factor *α* = 50. The temporal processing was uniform for all spatial levels and for all pixels within each level. The filtered spatial bands were then added back to the original signal and collapsed to generate the output video with magnified color.

We used the postprocessed videos to automatically extract eight facial patches (31 × 31 pixels) from each frame: two patches corresponding to two areas potentially affected by facial erythema (i.e., cheeks) and six corresponding to facial areas less prone to erythema, representing the background facial skin tone (forehead, philtrum, and nose) (Supplementary material ([Supplementary-material supplementary-material-1])). The location of the patches is detected all through the video, using an algorithm for the detection of facial landmarks. The red intensity value (red channel in the RGB color model, which is an integer that ranges from 0 to 255) in each of the 31 × 31 pixels of each patch was obtained and then averaged, to obtain a single red intensity value (*I*) per patch and frame. Blond and red-haired persons appear more erythematous because of reduced cutaneous melanin to obscure the erythema [16]. For that reason, the invisible facial redness for each subject and frame was calculated, for each of the cheeks (IFR_ch_), as the red intensity in the cheek (*I*_ch_) minus the background facial skin tone (*I*_B_), which was calculated for each subject and frame as the average of *I*_B1_, *I*_B2_, *I*_B3_, *I*_B4_, *I*_B5_, and *I*_B6_.

### 2.4. Statistical Analysis

Since the invisible facial redness increases and decreases periodically over time due to the heart pulsations, IFR values vary from frame to frame. Therefore, we used histograms to obtain a representation of the frequency distribution of the variable IFR measured for each cheek of each subject, throughout the video. We calculated the average IFR for each subject and cheek, over all frames. We used paired *t*-test to compare the average and standard deviation of IFR in the two cheeks of each subject. We compared the mean and standard deviation of IFR in diabetic vs. nondiabetic subjects, using Welch two-sample *t*-tests.

## 3. Results

**Figure 1:**
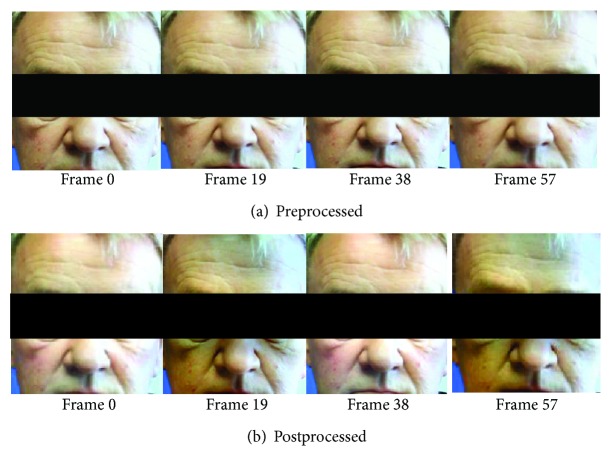
An example of a pre- and postprocessed video in a diabetic patient. Four frames from the original video sequence (a) and the same four frames with the subject's pulse signal amplified with magnification factor *α* = 100 (b).

The color magnification method applied to the videos recorded from all participants revealed the color changes caused by the blood flowing in and out of the face. An example of a pre- and postprocessed video of a diabetic patient is shown in Figure 1, including four frames from the original video sequence and the same four frames from the postprocessed video (magnification factor *α* = 100). No differences in color can be observed among the four frames of the preprocessed video. The postprocessed video, however, reveals a periodic variation in facial color, corresponding to the subject's pulse signal amplified. The postprocessed video can be used to approximate the subject's heartbeat. For instance, the time between the frames in Figure 1 is 0.38 s (19 frames in a video at 50 fps), i.e., 1 beat every 0.76 s, which results in an estimated heart rate for this patient of 79 beats per minute.

**Figure 2:**
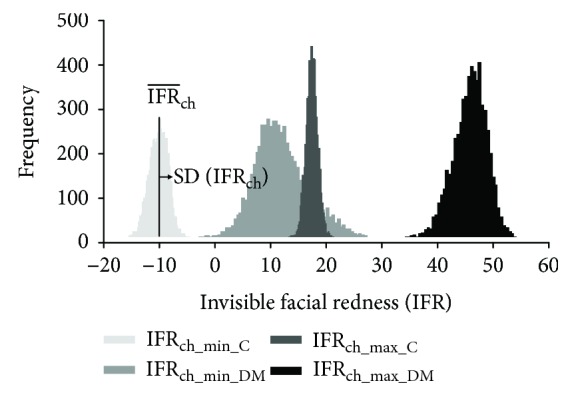
Histograms showing the frequency distributions of the invisible facial redness (IFR) at the two cheeks of one diabetic patient (the least reddish cheek, IFR_ch_min_DM_, and the most reddish cheek, IFR_ch_max_DM_) and the two cheeks of one nondiabetic subject (the least reddish cheek, IFR_ch_min_C_, and the most reddish cheek, IFR_ch_max_C_). In each distribution, the standard deviation, SD(IFR_ch_), measures how spread out the data is, whereas the arithmetic mean (${\bar{\text{IFR}}}_{\text{ch}}$) measures where the data is centered.

The histograms of IFR at the two cheeks of a diabetic patient and the two cheeks of one nondiabetic subject are shown in Figure 2, by way of example. Each histogram bin represents the number of times (all throughout a video segment) in which the IFR lies within each narrow range of IFR values. The higher the bar is, the greater the frequency of data values in that range. Therefore, the right tail and the left tail correspond to the least frequent redness intensities, that is, instances with the highest (e.g., frames 19 and 57 in Figure 1) and the lowest (e.g., frames 0 and 38 in Figure 1) redness intensity, while the center of the frequency distribution corresponds to more frequent, intermediate values of IFR.

The results from comparing the IFR at the two cheeks of all participants showed that the average facial redness at the right and left cheeks was significantly different (in repetition 1, *p* values of 0.028, 0.019, and 0.059, for segments 1, 2, and 3, respectively; and in repetition 2, *p* values of 0.040, 0.010, and 0.012, for segments 1, 2, and 3, respectively). On the contrary, the standard deviation of facial redness at the right and left cheeks was not significantly different (in repetition 1, *p* values of 0.410, 0.391, and 0.891, for segments 1, 2, and 3, respectively; and in repetition 2, *p* values of 0.855, 0.386, and 0.053, for segments 1, 2, and 3, respectively).

We then compared diabetic patients vs. controls in terms of average IFR at the cheeks with the highest and lowest redness intensities (IFR_ch_max_ and IFR_ch_min_) (Table 1). The average redness in the most reddish cheek of diabetic patients was higher in magnitude than the average redness in the most reddish cheek of control subjects (Table 1). Similarly, the average redness in the least reddish cheek of diabetic patients was higher in magnitude than the average redness in the least reddish cheek of controls. The results were consistent for all video segments and repetitions. However, these differences were not statistically significant. The remaining comparisons showed varying results.

On the contrary, we found that standard deviations yielded more systematic and significant results. The standard deviations of IFR in diabetic patients were significantly larger than in controls (3.81 vs. 2.30, *p* value < 0.001). This finding (i.e., standard deviations being larger in diabetic patients than in controls) was consistent across all repetitions and video segments, being statistically significant in the great majority of them (Table 2).

## 4. Discussion and Conclusions

Facial redness is related to noncutaneous diabetic complications [6, 9]. In routine clinical practice, facial redness of diabetic patients is evaluated based on subjective clinical observations of visible redness. In this paper, we have addressed the following questions: (1) are the faces of diabetic patients invisibly more reddish than the faces of nondiabetic subjects (even when this redness cannot be seen with the naked eye)?; (2) are the variations of invisible facial redness with the heartbeats different in diabetic patients compared to controls?; and (3) can we objectively quantify invisible facial redness and its periodic variations using a video recorded by a conventional digital camera? Our study showed that (1) even when facial redness is not visible for the clinician, invisible redness can be measured using a conventional digital camera and novel image processing methods; (2) faces of diabetic patients tend to be more “invisibly reddish” than those of controls, although these differences were not statistically significant; (3) as the heart beats and blood flows in and out of the face, all facial areas vary in color imperceptibly and periodically, between redder and paler, following a temporal frequency; and (4) this variation in facial redness due to the heart pulsation is consistently and significantly larger in diabetic patients compared to controls.

In our study, the two cheeks of each subject showed different average redness but not statistically different standard deviations of redness. In principle, the reason for the different average IFR at the two cheeks could be of physiological or of technical nature. From the physiological point of view, clinical findings of unilateral facial erythema in diabetic patients have not been reported in the literature, and they could not be either observed with the naked eye in none of our study participants. However, given that the color variations revealed with the image processing method cannot be seen with the naked eye, technically we are not able to completely exclude the possibility of unilateral invisible redness. Notwithstanding the foregoing, we believe that the reason for the differences in redness found between the right cheek and the left cheek is of technical nature. The color correction procedure performed on the captured facial image to decrease the effect of the different environment and illumination variations is applied to the entire image and is not able to compensate for different lighting conditions at different regions within a single image. The small differences in light on the two sides of the face and the presence of shades in one of the sides may introduce sources of error. Therefore, the main limitation of using the average redness (${\bar{\text{IFR}}}_{\text{ch}}$) as a marker for diabetic complications is the fact that, when illumination is not strictly controlled, the validity and reproducibility of the method are compromised.

The standard deviation of facial redness, however, is a more robust marker since it measures the data's width around its central value, and so it is not directly affected by systematic differences in illumination. Our study showed that the frequency distributions of IFR in diabetic patients were wider (i.e., larger standard deviations) than in controls. This can be interpreted that the variation of facial color at the cheeks with the heartbeats is significantly larger in diabetic patients compared to controls. The etiology of facial flushing has been recurrently described in the literature as a microangiopathic complication, which results in dilatation of superficial veins [4, 17]. However, given the innovative nature of our study, the etiology of the larger variation of facial redness in diabetic patients with the heartbeats is, at this point, unknown. Cutaneous dehydration and its spatial differences in diabetic patients may play a role, but at present, it is impossible to state the principal underlying cause for this finding.

Our study and the results derived from it are unprecedented. One limitation of the study is the small number of participants, and therefore, the current results, while encouraging, should be considered preliminary results demonstrating the proof of concept for invisible facial redness as a potential novel marker for diabetic complications. Our study sets the stage for a clinical trial of sufficient size and duration, with appropriate adjustment for confounders, and that should include patients diagnosed with nephropathy, retinopathy, and/or neuropathy, diabetic patients prior to the diagnosis of these complications, prediabetic patients, nondiabetic members of diabetic families, and nondiabetic controls with no family history of DM. Confirmation of our results would lay the foundation to provide the patients and the healthcare sector with a feasible, noninvasive, and cost-effective telemedicine tool that allows remote monitoring and continuous risk assessment of diabetic patients and automatic detection of early signs of diabetic complications, by image processing of facial videos captured at home with the patient's smartphone.

## Figures and Tables

**Table 1:** Comparison of average invisible facial redness (${\bar{\text{IFR}}}_{\text{ch}}$) in diabetic patients and nondiabetic controls, where IFR_ch_min_ is the IFR at the least reddish check of each subject and IFR_ch_max_ is the IFR at the most reddish check of each subject. Rep = repetition; Seg = segment of the video.

Rep	Seg	Averages				Comparisons			
		Control subjects (C)		Diabetic patients (DM)		${\bar{\text{IFR}}}_{\text{ch}\_\min\_\text{C}}$ vs. ${\bar{\text{IFR}}}_{\text{ch}\_\min\_\text{DM}}$	${\bar{\text{IFR}}}_{\text{ch}\_\max\_\text{C}}$ vs. ${\bar{\text{IFR}}}_{\text{ch}\_\max\_\text{DM}}$	${\bar{\text{IFR}}}_{\text{ch}\_\max\_\text{C}}$ vs. ${\bar{\text{IFR}}}_{\text{ch}\_\min\_\text{DM}}$	${\bar{\text{IFR}}}_{\text{ch}\_\min\_\text{C}}$ vs. ${\bar{\text{IFR}}}_{\text{ch}\_\max\_\text{DM}}$
		${\bar{\text{IFR}}}_{\text{ch}\_\min}$	${\bar{\text{IFR}}}_{\text{ch}\_\max}$	${\bar{\text{IFR}}}_{\text{ch}\_\min}$	${\bar{\text{IFR}}}_{\text{ch}\_\max}$	*p* value	*p* value	*p* value	*p* value
R1	A	7.7	25.3	13.2	32.5	0.347	0.146	0.029	<0.001
	B	7.0	24.0	11.1	31.8	0.520	0.138	0.036	<0.001
	C	4.9	23.5	8.5	29.2	0.571	0.276	0.012	0.001

R2	A	5.6	24.7	9.8	29.2	0.472	0.392	0.016	<0.001
	B	5.3	23.5	6.7	29.2	0.806	0.286	0.006	<0.001
	C	4.7	23.3	5.7	30.1	0.876	0.237	0.008	<0.001

**Table 2:** Comparison of standard deviations (SD) of invisible facial redness (IFR) in diabetic patients and nondiabetic controls, where ch_min is the least reddish check of each subject and ch_max is the most reddish check of each subject. Rep = repetition; Seg = segment of the video.

Rep	Seg	Standard deviations				Comparisons			
		Control subjects (C)		Diabetic patients (DM)		SD(IFR_ch_min_C_) vs. SD(IFR_ch_min_DM_)	SD(IFR_ch_max_C_) vs. SD(IFR_ch_max_DM_)	SD(IFR_ch_max_C_) vs. SD(IFR_ch_min_DM_)	SD(IFR_ch_min_C_) vs. SD(IFR_ch_max_DM_)
		SD(IFR_ch_min_)	SD(IFR_ch_max_)	SD(IFR_ch_min_)	SD(IFR_ch_max_)	*p* value	*p* value	*p* value	*p* value
R1	A	2.44	2.05	3.66	3.87	0.001	0.027	0.001	0.018
	B	2.71	2.45	3.62	3.59	0.090	0.124	0.053	0.185
	C	2.24	1.89	4.09	3.67	0.003	0.016	0.004	0.019

R2	A	2.51	2.38	3.95	3.93	0.037	0.027	0.026	0.041
	B	2.52	2.11	3.74	3.44	0.021	0.021	0.003	0.105
	C	2.44	1.92	4.34	3.63	0.002	0.025	0.005	0.037

## Data Availability

The data used to support the findings of this study are available from the corresponding author upon request.

## Supplementary Materials

Supplementary Materials Supplementary material includes a picture that indicates the location of the eight facial patches (31 × 31 pixels) from each frame: two patches corresponding to two areas potentially affected by facial erythema (i.e., cheeks) and six corresponding to facial areas less prone to erythema, representing the background facial skin tone (forehead, philtrum, and nose).

## References

[1] Harding J. L., Pavkov M. E., Magliano D. J., Shaw J. E., Gregg E. W. (2019). Global trends in diabetes complications: a review of current evidence. *Diabetologia*.

[2] Liew G., Wong V. W., Ho I. V. (2017). Mini review: changes in the incidence of and progression to proliferative and sight-threatening diabetic retinopathy over the last 30 years. *Ophthalmic Epidemiology*.

[3] Litwak L., Goh S. Y., Hussein Z., Malek R., Prusty V., Khamseh M. E. (2013). Prevalence of diabetes complications in people with type 2 diabetes mellitus and its association with baseline characteristics in the multinational A1chieve study. *Diabetology and Metabolic Syndrome*.

[4] Bustan R. S., Wasim D., Yderstræde K. B., Bygum A. (2017). Specific skin signs as a cutaneous marker of diabetes mellitus and the prediabetic state - a systematic review. *Danish Medical Journal*.

[5] Murphy-Chutorian B., Han G., Cohen S. R. (2013). Dermatologic manifestations of diabetes mellitus. *Endocrinology and Metabolism Clinics of North America*.

[6] Levy L., Zeichner J. A. (2012). Dermatologic manifestation of diabetes. *Journal of Diabetes*.

[7] Kamel M. I., Elhenawy Y. I., Saudi W. M. (2018). Relation between cutaneous and extracutaneous complications in pediatric patients with type 1 diabetes. *Dermato-Endocrinology*.

[8] Paron N. G., Lambert P. W. (2000). Cutaneous manifestations of diabetes mellitus. *Primary Care*.

[9] Ngo B. T., Hayes K. D., DiMiao D. J., Srinivasan S. K., Huerter C. J., Rendell M. S. (2005). Manifestations of cutaneous diabetic microangiopathy. *American Journal of Clinical Dermatology*.

[10] Pavlovic M. D., Milenkovic T., Dinic M. (2007). The prevalence of cutaneous manifestations in young patients with type 1 diabetes. *Diabetes Care*.

[11] Demirseren D. D., Emre S., Akoglu G. (2014). Relationship between skin diseases and extracutaneous complications of diabetes mellitus: clinical analysis of 750 patients. *American Journal of Clinical Dermatology*.

[12] Gitelson S., Wertheimer-Kaplinski N. (1965). Color of the face in diabetes mellitus: observations on a group of patients in Jerusalem. *Diabetes*.

[13] Durand F., Freeman W. T., Rubinstein M. (2014). A world of movement. *Scientific American*.

[14] Wadhwa N., Freeman W. T., Durand F. (2016). Eulerian video magnification and analysis. *Communications of the ACM*.

[15] Wu H.-Y., Rubinstein M., Shih E., Guttag J., Durand F., Freeman W. (2012). Eulerian video magnification for revealing subtle changes in the world. *ACM Transactions on Graphics*.

[16] Ferringer T., Miller O. F. (2002). Cutaneous manifestations of diabetes mellitus. *Dermatologic Clinics*.

[17] Duff M., Demidova O., Blackburn S., Shubrook J. (2015). Cutaneous manifestations of diabetes mellitus. *Clinical Diabetes*.

