# Supplementary material for: Invisible Color Variations of Facial Erythema: A Novel Early Marker for Diabetic Complications?
Source: J Diabetes Res. 2019 Sep 2;2019:4583895. doi: 10.1155/2019/4583895 (PMC6745171; doi:10.1155/2019/4583895)
Supplement: Supplementary Materials — Supplementary material includes a picture that indicates the location of the eight facial patches (31 × 31 pixels) from each frame: two patches corresponding to two areas potentially affected by facial erythema (i.e., cheeks) and six corresponding to facial areas less prone to erythema, representing the background facial skin tone (forehead, philtrum, and nose). [file 4583895.f1.docx]

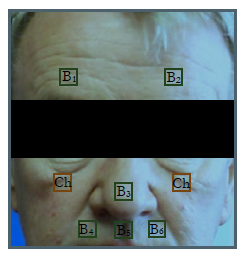


Figure 1. Location of the eight patches, two corresponding to areas potentially affected by facial erythema (Ch) and six corresponding to the background facial skin tone (B_1_ to B_6_)
